# Investigating the Utility of the SOFA Score and Creating a Modified SOFA Score for Predicting Mortality in the Intensive Care Units in a Tertiary Hospital in Jordan

**DOI:** 10.1155/2023/3775670

**Published:** 2023-08-07

**Authors:** Anas H. A. Abu-Humaidan, Fatima M. Ahmad, Laith S. Theeb, Abdelrahman J. Sulieman, Abdelkader Battah, Amjad Bani Hani, Mahmoud Abu Abeeleh

**Affiliations:** ^1^Department of Pathology, Microbiology, and Forensic Medicine, School of Medicine, The University of Jordan, Amman, Jordan; ^2^Department of Clinical Sciences, School of Science, The University of Jordan, Amman, Jordan; ^3^Department of General Surgery, Section of Cardiovascular Surgery, Jordan University Hospital, Amman, Jordan

## Abstract

**Background:**

The utility of the Sequential Organ Failure Assessment (SOFA) score in predicting mortality in the intensive care unit (ICU) has been demonstrated before, but serial testing in various settings is required to validate and improve the score. This study examined the utility of the SOFA score in predicting mortality in Jordanian ICU patients and aimed to find a modified score that required fewer laboratory tests.

**Methods:**

A prospective observational study was conducted at Jordan University Hospital (JUH). All adult patients admitted to JUH ICUs between June and December 2020 were included in the study. SOFA scores were measured daily during the whole ICU stay. A modified SOFA score (mSOFA) was constructed from the available laboratory, clinical, and demographic data. The performance of the SOFA, mSOFA, qSOFA, and SIRS in predicting ICU mortality was assessed using the area under the receiver operating characteristic curve (AUROC).

**Results:**

194 patients were followed up. SOFA score (mean ± SD) at admission was significantly higher in non-survivors (7.5 ± 3.9) compared to survivors (2.4 ± 2.2) and performed the best in predicting ICU mortality (AUROC = 0.8756, 95% CI: 0.8117–0.9395) compared to qSOFA (AUROC = 0.746, 95% CI: 0.655–0.836) and SIRS (AUROC = 0.533, 95% CI: 0.425–0.641). The constructed mSOFA included points for the hepatic and CNS SOFA scores, in addition to one point each for the presence of chronic kidney disease or the use of breathing support; it performed as well as the SOFA score in this cohort or better than the SOFA score in a subgroup of patients with heart disease.

**Conclusion:**

SOFA score was a good predictor of mortality in a Jordanian ICU population and better than qSOFA, while SIRS could not predict mortality. Furthermore, the proposed mSOFA score which employed fewer laboratory tests could be used after validation from larger studies.

## 1. Introduction

Clinical scoring systems provide a helpful tool for predicting the outcome of patients in critical care and usually derive a severity score from a variety of measurable clinical and laboratory variables [1]. In addition to prognostication and evaluation of patient status, scoring systems are frequently used in the assessment of various treatments and interventions, as well as policymaking, resource allocation, and quality of care [2, 3]. The Sequential Organ Failure Assessment (SOFA), Quick SOFA (qSOFA), Acute Physiology and Chronic Health Evaluation (APACHE), Simplified Acute Physiology Score (SAPS), and Mortality Probability Model (MPM0) are some of the most widely used scoring systems [1]. Although several systems have been proposed, each with its strengths and weaknesses, a highly sensitive and specific test for risk stratification of mortality among ICU patients is still lacking [4, 5].

The SOFA scoring scheme assigns 1 to 4 points for each organ system (respiratory, circulatory, renal, coagulation, hepatic, and central nervous systems) depending on the level of dysfunction, where a score of 0 is given for normal function while 4 is given for severe dysfunction [6]. Although the SOFA score was initially developed to continuously evaluate organ dysfunction in septic patients, it could be similarly applied to non-septic patients [7], as the risk of ICU mortality is highly correlated with organ dysfunction [8]. For rapid assessment of organ failure, a relatively recent qSOFA score was developed by assigning 1 point each for a systolic blood pressure less than or equal to 100 mm·Hg; a respiratory rate greater than or equal to 22 breaths/min; and any alteration in mental status [7]. On the other hand, SIRS is characterized but not limited to more than one of the following manifestations: (1) a body temperature >38°C or <36°C; (2) a heart rate >90 beats per minute; (3) a respiratory rate >20 breaths per minute, or hyperventilation, as indicated by a PaCO_2_ of <32 mm Hg; and (4) a leukocyte count of >12000 or <4000/microliter or over 10% immature forms or bands [9].

Several factors can affect the discriminative ability of scoring systems such as the setting (organization, policies, and staff experience) and the population under investigation. When considering in-hospital mortality, for example, the discriminative ability of the SOFA score was greater than qSOFA or SIRS in a large study in Australian and New Zealand ICUs [10], but such findings cannot be extended to the emergency department or ward admissions in low- and middle-income countries (LMICs). Additionally, variables unique to the population tested such as comorbidities should be considered; in Jordan, for example, the prevalence of diabetes is around 4 times that in Australia [11], and diabetics have unique pathologies that could affect the performance of predictive scores [12]. Finally, advancements in the medical field and shifts in prevailing diseases further conclude the need for serial testing of predictive scores.

Therefore, this study aimed to assess the use of the SOFA score in predicting mortality in Jordanian ICU patients, for which no previous data were found. The study also compared the predictive ability of the SOFA score to qSOFA and SIRS scores. Those scores were chosen for comparison because they require no additional clinical or lab variables than those routinely collected for all ICU patients, they are commonly used in the ICU [13], and the evidence from LMIC on their predictive ability is conflicting [14]. Daily SOFA scores and other relevant variables were recorded and compared in ICU survivors and non-survivors. SOFA, qSOFA, and SIRS predictive ability were assessed in the whole cohort and various subgroups of patients using the area under the receiver operating characteristic curve (AUROC) analysis. In addition, a modified SOFA score (mSOFA) that used fewer laboratory tests was also constructed and assessed for its ability to predict ICU mortality.

## 2. Methods

### 2.1. Study Design and Population

This was a single-center prospective cohort study conducted at the adult ICUs of Jordan University Hospital (JUH), Amman, which is the largest teaching tertiary hospital in the capital and serves thousands of patients from various regions of the country. We followed up 194 admissions to JUH ICUs over a period of 6 months (from July 15, 2020, through January 15, 2021). The characteristics of the study cohort were described previously in a study that investigated the characteristics of adult sepsis patients in the ICU [15]. Inclusion criteria included all patients who were ≥18 years admitted to JUH's adult ICUs within the study period. The only exclusion criterion was being under the age of 18. The bed capacity of JUH is about 600, with approximately 32 beds distributed to 3 adult ICUs: surgical, medical, and anesthesia. In 2020, these ICUs served 635 admissions according to the hospital's medical care department.

### 2.2. Ethical Approval

The Institutional Review Board (IRB) approved the study protocol at JUH (Ref. No. 189/2020). In addition, the work was conducted according to the principles of Good Clinical Practice (GCP) that have their origin in the Declaration of Helsinki (64th World Medical Association General Assembly, Fortaleza, Brazil, October 2013). All collected data were treated with confidentiality.

Participation in the study was voluntary. After fully explaining the study objectives, written and signed informed consent was obtained from all conscious patients who agreed to participate. Assessing the level of consciousness involved checking orientation: participants who were able to promptly and spontaneously state their name, location, and date or time were said to be conscious. For patients who were unconscious or unable to consent at the time of admission, consent was obtained from first-degree relatives. However, consent was sought from those who survived once they regained consciousness or improved clinically to a stage where they can consent.

### 2.3. Data Collection and Definitions

The recorded data were categorized into demographic, clinical, and laboratory variables for each admission. Demographic variables included age, sex, height, weight, comorbidities, date of admission to the hospital and ICU, and date of discharge from the hospital and ICU.

Clinical variables included the ICU section (divided into SICU, MICU, and AICU), source of ICU admission, reasons for admission, origin of infection for patients with sepsis, vital signs on admission, and medical interventions (mechanical ventilation, catheterization, and blood transfusion).

Laboratory variables included hemoglobin, packed cell volume (PCV), total WBC count, neutrophils, lymphocytes, platelet count, creatinine, bilirubin, arterial oxygen partial pressure (PaO_2_), fractional inspired oxygen (FiO_2_), total protein, random blood sugar (RBS), and electrolytes (Na, K, and CL) as well as microbiological findings such as culture results and type of samples used for culture.

ICU mortality was defined as death at any time of the ICU stay. ICU length of stay (LOS) was calculated as (the date of ICU discharge–the date of ICU admission). Hospital LOS was calculated as the discharge date–the hospital admission date. The diagnosis of sepsis was based on the diagnostic guidelines of Sepsis-3 that were set in 2016 by the *Third International Consensus Definitions for Sepsis and Septic Shock* [7]. Sepsis was defined as a suspected or documented infection plus an acute organ dysfunction represented by an increase in SOFA score equal to or greater than 2 points. Criteria for the SOFA score, qSOFA, and SIRS can be found in the supplementary material (Supplementary Tables 1–3).

The *mean* SOFA was the average of daily SOFA scores of any individual during their ICU stay; *maximum* SOFA was the highest SOFA score of any individual during their ICU stay; and *delta* SOFA was the SOFA score after 48 hours of admission–SOFA score at admission. In the mSOFA score, chronic kidney disease was defined as structural or functional abnormalities of the kidneys for ≥3 months, and dialysis patients were included only if they fulfilled the aforementioned definition [16]. Breathing support included a simple face mask, nasal cannula, non-rebreathing mask, venturi mask, CPAP, BiPAP, or intubation.

### 2.4. Data Analysis and Prediction Model Assessment

Data generated were organized in Microsoft Excel and statistical analysis was done using R statistical language, version 4.1.3 (R Foundation for Statistical Computing, Vienna, Austria). Descriptive statistics were presented as counts/percentages for categorical variables and as means ± standard deviation/medians for continuous variables. The association between survival status and different scores was assessed using Student's *t*-test for continuous variables, while the Wilcoxon rank-sum test was used when normality was violated. Normality was tested using histograms and the Shapiro–Wilk test. For categorical variables, the chi-square test and Fisher's exact test were used.

The association of the SOFA score and SOFA organ-specific subcomponents to SIRS was assessed visually by producing scatter plots and quantitatively using Spearman's correlation coefficient. Odds ratios (ORs) were calculated using univariate and multivariate logistic regression models. Backward logistic regression was used to reach the minimum number of significant variables for the predictive model. The area under the receiver operating characteristic curve (AUROC) was used to assess the discriminative power of SOFA, qSOFA, SIRS, and the modified SOFA models. An AUROC of 0.5 indicates that the model has no discriminative power and an AUROC of 1.0 indicates perfect discriminative power [17]. De Long et al.'s method was used to test the statistical significance of the difference between AUROC curves [18]. All tests were 2-sided, and a *p* value of less than 0.05 was considered statistically significant.

## 3. Results

### 3.1. SOFA, qSOFA, and SIRS Scores of Survivors and Non-Survivors

Demographic and clinical data of the 194 patients admitted to the adult ICUs at JUH during the study period were described in a previous epidemiological study about sepsis in the ICU [15]. In brief, the average age in the cohort was 60 ± 16 years, with 107 (55.2%) males and 87 (44.8%) females and an all-cause ICU mortality rate of 18.0%. Forty-five patients (23.2%) had sepsis during their ICU stay according to the Sepsis-3 criteria. The complete list of demographic, clinical, and laboratory variables used in this study can be found in the supplementary material (Supplementary Table 4).

The SOFA score at admission among the study population was 3.3 ± 3.21 and was significantly higher in non-survivors than in survivors (7.4 ± 3.84 vs. 2.4 ± 2.2, respectively, *p* < 0.001). Organ-specific subcomponents of the SOFA scores (referred to herein as respiratory SOFA, coagulation SOFA, hepatic SOFA, cardiovascular SOFA, CNS SOFA, and renal SOFA) which reflect the dysfunction in each organ system separately were significantly higher in non-survivors (Table 1). Similarly, a significant difference was found between survivors and non-survivors in fulfilling 2 or more of the qSOFA criteria (Table 1). On the other hand, having SIRS (fulfilling 2 or more SIRS criteria) was similar in survivors and non-survivors with 37.1% and 37.8%, respectively, satisfying SIRS criteria at admission (Table 1).

Examination of the following SOFA score derivatives: the *mean* SOFA (average of daily SOFA scores of any individual during their ICU stay); *maximum* SOFA (highest SOFA score of any individual during their ICU stay); and *delta* SOFA (SOFA score after 48 hours of admission–SOFA score at admission), showed significantly higher scores in non-survivors than in survivors (Table 1).

**Figure 1:**
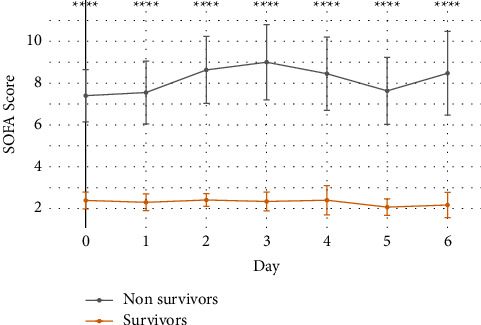
Serial measurement of SOFA scores in ICU patients. The graph represents daily SOFA score measurements in survivors (yellow line) and non-survivors (grey line) during the first week of admission to the ICU. Circles represent the mean while whiskers represent the standard deviation. ^*∗∗∗∗*^*p* < 0.001.

The serial measurement of the SOFA score across the ICU stay allowed for visualization of the change in scores for survivors and non-survivors over the first 7 days (Figure 1). Scores of both survivors and non-survivors were relatively constant from admission to discharge or death, and the highest SOFA score for survivors and non-survivors was on day 2 and day 3, respectively. There was a significant difference between survivors' and non-survivors' scores during each day of the first 7 days (*p* < 0.001).

Since the criteria of SIRS should reflect a severe inflammatory state, we examined whether an organ system failure is associated with SIRS development, and Spearman's correlation coefficient (*r*_*s*_) was used to investigate a correlation between each organ system SOFA and SIRS criteria (scored 0–4). SOFA and organ-specific SOFA scores were not correlated with SIRS (*p* > 0.05 for all SOFA subscores) (Supplementary Figure 1).

### 3.2. Building a Modified SOFA Score

To derive a useful score using minimum yet logical clinical and laboratory requirements, a univariate logistic regression analysis of all potential predictors of mortality in the ICU was conducted. The analysis included vital signs, lab tests, organ-specific SOFA scores, comorbidities, and breathing support at admission. All organ-specific SOFA scores were highly significant as adverse predictors of ICU mortality, as well as chronic kidney disease (CKD) and atrial fibrillation, while lab tests included creatinine and potassium (Supplementary Table 5).

**Figure 2:**
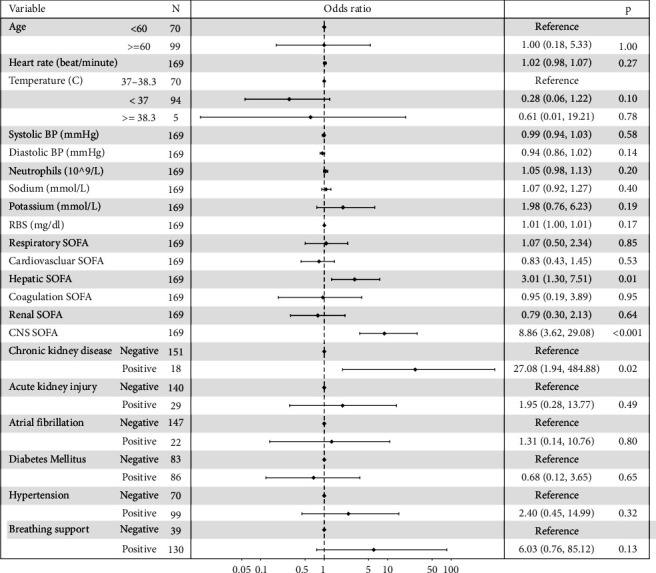
A forest plot based on multivariate analysis of clinical and laboratory results related to ICU mortality. Following univariate analysis of all potential predictors of mortality in the ICU, variables with a *p* value ≤0.25 were first examined for multicollinearity and then used for the multivariate analysis.

Afterward, significant variables with a *p* value ≤0.25 in univariate analysis were examined for multicollinearity, where a variance inflation factor (VIF) of 4 or more was sufficient to remove the variable [19]. Lab results reflecting any of the organ-specific SOFA scores were excluded from multivariate analysis due to multicollinearity issues, for example, creatinine levels were excluded since they reflect the renal SOFA score. Variables considered for multivariate logistic regression included age, heart rate, body temperature, systolic and diastolic blood pressure, neutrophils, sodium, potassium, random blood sugar, organ-specific SOFA scores, CKD, AKI, atrial fibrillation, diabetes, hypertension, and breathing support. Finally, multivariate analysis of patients revealed that hepatic SOFA, CNS SOFA, and CKD were independent risk factors for ICU mortality (Figure 2).

Subsequently, a backward logistic regression analysis of all variables considered for multivariate analysis was performed to end up with the most reduced predictive model, and a modified SOFA (mSOFA) score was constructed (Table 2). Hepatic SOFA and CNS SOFA were graded (0–4) each, the same as in the SOFA scoring system. The presence of chronic kidney disease (CKD) as a comorbidity and breathing support at admission were given 1 point each. The cohort had a mean mSOFA of 1.58 ± 1.3 (range, 0–10), and there was a significant difference in mSOFA between survivors and non-survivors (1.19 in survivors vs. 3.20 in non-survivors, *p* value <0.001). All patients with an mSOFA score above 4 at admission died in the ICU.

### 3.3. Mortality Prediction Using SOFA, qSOFA, SIRS, and mSOFA

To assess the performance of different scores in predicting ICU mortality, an AUROC analysis was performed.

**Figure 3:**
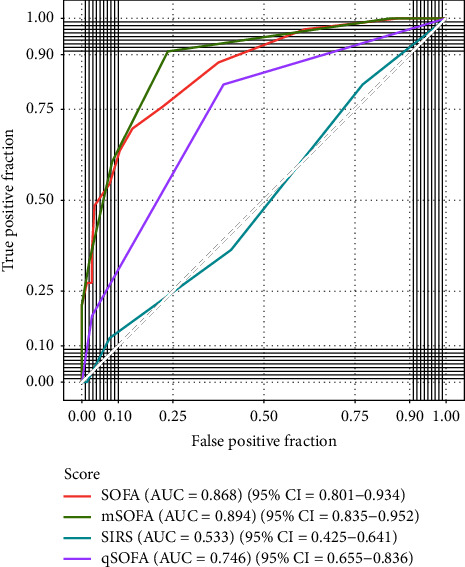
Receiver operating characteristic (ROC) curves for different scores at admission. The area under the curve (AUC) for each ROC curve is presented in the legend on the right of the graph along with the 95% confidence intervals (CIs). AUC quantifies the discriminative ability of each model.

The analysis revealed that both SOFA and mSOFA scores had the best predictive ability (AUROC = 0.868, 95% CI: 0.801–0.934) and (AUROC = 0.894, 95% CI: 0.835–0.952), respectively, with qSOFA behind (AUROC = 0.746, 95% CI: 0.655–0.836), and SIRS performed the worst (AUROC = 0.533, 95% CI: 0.425–0.641) (Figure 3).

Specificity and sensitivity as well as cutoff values for each score are found in the supplementary material (Supplementary Table 6). No statistically significant difference in the predictive ability of SOFA and mSOFA was found, but both scores were superior to qSOFA and SIRS (Supplementary Table 7).

AUROC values were also measured for the SOFA score derivatives collected over the ICU stay. The *maximum* SOFA had the best AUROC values (AUROC = 0.966, 95% CI: 0.928–1.000), followed by *average* SOFA (AUROC = 0.954, 95% CI: 0.912–0.996), while *delta* SOFA performed worse than SOFA at admission (AUROC = 0.626, 95% CI: 0.506–0.745) (Supplementary Figure 2).

### 3.4. Mortality Prediction in Various Subgroups of ICU Patients

**Figure 4:**
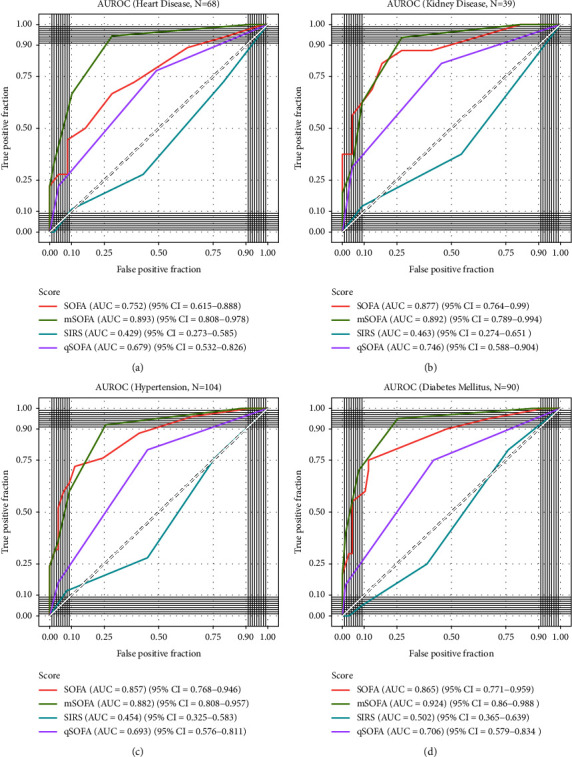
Receiver operating characteristic (ROC) curves for different scores at admission in different subgroups of patients. The area under the curve (AUC) for each ROC curve is presented in the legend on the right of each graph along with the 95% confidence intervals (CIs). AUC quantifies the discriminative ability of each model. The discriminative ability was tested in different subgroups of patients including patients with (a) heart disease, (b) kidney disease, (c) hypertension, and (d) diabetes mellitus.

The discriminative ability of SOFA, mSOFA, qSOFA, and SIRS was tested in four subgroups of patients, and the division was done based on the most common comorbidities found in this cohort, which were heart disease (*n* = 68), kidney disease (*n* = 39), hypertension (*n* = 104), and diabetes mellitus (*n* = 90) (Figure 4).

mSOFA performed significantly better than qSOFA and SIRS in predicting mortality in all 4 subgroups of patients; in addition, mSOFA was superior to SOFA in predicting mortality among patients with heart disease (AUROC, 0.893 vs. 0.752, respectively, *p*=0.033), while SOFA performed significantly better than qSOFA and SIRS in patients with kidney injury, hypertension, and diabetes (Supplementary Table 8).

## 4. Discussion

This study investigated the utility of using the SOFA score in predicting mortality in the ICUs of a large tertiary hospital in Jordan, a setting for which no previous studies were found. The demographic, clinical, and laboratory data collected from patients during their ICU stay were also used for the construction of an easily applicable prediction score that required fewer laboratory results than the SOFA score. This ICU cohort was followed up daily for a period of 6 months, and its characteristics with regard to the development of sepsis were described in a previous study [15].

The ICU non-survivors in this study had significantly higher SOFA scores on admission than survivors with a median of 7.5 points, which is comparable to a study from India where the average day 1 SOFA score for non-survivors was around 7.5 as well, although the total mortality of the cohort was higher at 39% and the cohort was smaller with 44 patients [20]. Notably, the population of the ICU affects the relationship between admission SOFA score and mortality. For example, in contrast to this study, a recent study conducted on severe COVID-19 patients admitted to the ICU showed that the admission mean SOFA score of non-survivors was 5.2, which was not significantly different from survivors who had a mean score of 4.3 [21].

The SOFA score was previously shown to be a good predictor of ICU mortality in several recent studies [21–23], mostly from high-income countries and usually conducted in resource-rich settings. This study showed that the SOFA score was good in predicting ICU mortality in an LMIC and was superior to both qSOFA and SIRS with an AUROC of 0.868. The good performance of the SOFA score in our study was comparable to studies from high-income countries which usually report an AUROC between 0.61 and 0.88 [24]. The utility of the SOFA score was further confirmed by serial SOFA score measurements, which had the best AUROC values with a *maximum* SOFA and an *average* SOFA AUROC of 0.966 and 0.954, respectively, and such values are in line with previous reports [25, 26], while the change in the SOFA score within the first 48 hours, or *delta* SOFA, had a worse AUROC (0.626) than the initial SOFA. It should be noted that delta SOFA can have more than one definition; many studies define it as the (total maximum SOFA-admission SOFA) [27, 28]. Our study had a fixed day definition of delta sofa (48 hours SOFA–admission SOFA), which could have affected its predictive ability. Nevertheless, a systematic review of 11 studies indicated that the best AUROC values were usually reported for max SOFA (range = 0.792 to 0.922) and the lowest for delta SOFA (range = 0.51 to 0.828) [24].

The dependence of qSOFA on clinical characteristics rather than laboratory tests makes it favorable in emergency department (ED) settings [29] and in resource-limited settings where not all laboratory tests are available [30]. This study indicated that while qSOFA scores were significantly different in survivors and non-survivors, they did not predict mortality as well as SOFA scores in ICU patients. The AUROC value of qSOFA in this study was 0.746, which is comparable to a study performed on severe trauma patients at the ED, although the population in that study had a more severe disease [29]. A study comparing qSOFA and SOFA in an LMIC showed that qSOFA was better at predicting mortality in sepsis patients, with AUROC of 0.92 and 0.63 for qSOFA and SOFA, respectively [14]. The results are contrary to the results of this study, probably because only 23.2% of patients in this study had sepsis.

The use of SIRS positivity on admission to predict mortality has had varying results. A Brazilian study of ICU patients concluded that although SIRS development was associated with mortality, it was a worse predictor of mortality compared to SAPS-3 [31]. A recent meta-analysis of clinical predictive scores in LMIC showed high sensitivity of SIRS in predicting mortality in sepsis patients [14]. On the other hand, this study demonstrated that having SIRS on admission was similar in survivors and non-survivors and indicated that SIRS could not predict mortality with an AUROC of 0.533. Furthermore, no correlation was found between the development of SIRS and the failure of an organ system as measured by the SOFA score. Development of SIRS was common in all ICU patients on admission with a prevalence of 37.6% in this study, but it was not useful in the prognostication of ICU patients.

Using the cohort in this study, a modified SOFA score was generated depending on the most predictive variables of mortality. The mSOFA score used hepatic SOFA, CNS SOFA, CKD, and breathing support with a total of 10 points. ICU non-survivors had a significantly higher mSOFA at admission than survivors and all patients with a score above 4 at admission died in the ICU. Additionally, the mSOFA had equivalent ICU mortality prediction to regular SOFA, although it utilized fewer laboratory tests. The respiratory SOFA was replaced by only one point for using any type of breathing support, and renal SOFA was replaced by having documented chronic kidney disease, while the coagulation SOFA was disregarded. This meant that laboratory tests for arterial oxygen, creatinine, and platelet count were not needed. This could be useful for the serial measurement of SOFA, especially in situations such as pandemics where ICUs may be overwhelmed and resources are scarce. Several studies looked at modifications in the SOFA score for better mortality prediction using fewer variables; one study indicated that a modified SOFA that replaced hepatic SOFA with scleral icterus or jaundice and disregarded coagulation SOFA predicted mortality as well as the standard SOFA (SOFA AUC = 0.83; mSOFA AUC = 0.84) [32]. The same modified SOFA from that study was applied in Iran and showed similar predictive ability to SOFA (AUCs of 0.751 and 0.739, respectively) [5], which could indicate the feasibility of using the mSOFA from this study in different settings after further validation.

The predictive ability of the SOFA score was measured in various groups of participants, and the presence of comorbidities led to different prediction values with an AUROC of 0.752 in patients with heart diseases to 0.877 in patients with kidney diseases. A study with a large cohort of patients admitted to the ICU following cardiac surgery revealed that day 1 SOFA was able to predict mortality with a comparable AUROC of 0.809 to this study but concluded that in that specific group of ICU patients, traditional scores such as APACHE-IV and SAPS-II were better [33]. Patients who had heart disease in this study were the only group where the mSOFA had a better predictive ability than the regular SOFA. In all other groups, the SOFA score and mSOFA both had good predictive ability with no significant differences.

This study had some limitations that should be considered. Firstly, it was performed on ICU patients in a single hospital, and while JUH is the largest tertiary hospital in the capital and receives patients from various regions of the country, it would be difficult to generalize the utility of the SOFA score to other ICUs in Jordan which vary greatly in their capabilities. Another limitation is related to the validation of the mSOFA score, which was only done internally in various subgroups of patients and would require larger multicenter cohorts' validation before clinical use. Finally, although mSOFA here might provide a suitable alternative to the standard SOFA, it is only useful in post-admission settings. Since mSOFA in this study uses breathing support as one of the criteria, it cannot be used in the pre-admission triage of patients.

In conclusion, SOFA score utility and superiority to qSOFA and SIRS scores in predicting mortality in the ICU were confirmed in a Jordanian population, adding to the scarce data from developing countries. Furthermore, it indicated the possibility of using a modified score for ICU patients that uses fewer laboratory tests and instead depends on clinical characteristics.

## Figures and Tables

**Table 1:** SOFA, qSOFA, and SIRS scores of survivors and non-survivors.

Scores^a^	Cohort (*n* = 194)	Survivors (*n* = 159)	Non-survivors (*n* = 35)	*p* value^b^
*At admission*				
Total SOFA	3.3 ± 3.21 (2.31)	2.4 ± 2.2 (1.9)	7.4 ± 3.84 (7.5)	**<0.001**
Respiratory SOFA	1.3 ± 1.04 (1.27)	1.2 ± 0.93 (1.17)	1.9 ± 1.28 (1.88)	**0.002**
Coagulation SOFA	0.2 ± 0.53 (0.14)	0.1 ± 0.44 (0.1)	0.4 ± 0.78 (0.32)	**0.003**
Hepatic SOFA	0.3 ± 0.71 (0.22)	0.3 ± 0.63 (0.18)	0.6 ± 0.95 (0.41)	**0.018**
Cardiovascular SOFA	0.7 ± 1.38 (0.25)	0.4 ± 1.13 (0.15)	1.8 ± 1.83 (1)	**<0.001**
CNS SOFA	0.4 ± 0.88 (0.22)	0.1 ± 0.4 (0.1)	1.5 ± 1.48 (1.08)	**<0.001**
Renal SOFA	0.5 ± 1.01 (0.31)	0.3 ± 0.81 (0.22)	1.3 ± 1.41 (0.95)	**<0.001**
qSOFA ≥ 2	11 (5.7%)	4 (2.5%)	7 (20%)	**0.001**
SIRS ≥ 2	73 (37.6%)	60 (37.7%)	13 (37.1%)	0.948

*During the ICU stay*				
Mean SOFA	3.2 ± 3.37 (2.01)	2 ± 1.53 (1.62)	8.9 ± 3.64 (9.89)	**<0.001**
Maximum SOFA	4.5 ± 4.32 (2.86)	2.9 ± 2.36 (2.4)	11.9 ± 3.53 (12.25)	**<0.001**
Delta SOFA	0.0685 ± 1.87 (0.0)	−0.330 ± 1.55 (0.0)	1.55 ± 2.22 (1.0)	**<0.001**

^a^Scores are presented as mean ± SD (median) or count (percent). ^b^*p* values ≤0.05 are in bold.

**Table 2:** Variables of the mSOFA and the points assigned to them.

Variable	OR	CI (95%)	*P* value	Points assigned
Hepatic SOFA^1^	2.724	1.33–5.56	0.006	0–4
CNS SOFA^1^	9.497	3.79-23.8	<0.001	0–4
Chronic kidney disease^2^	15.261	3.26-71.53	0.001	0-1
Breathing support^3^	8.449	1.36-52.64	0.022	0-1

^1^The same SOFA criteria are used here; ^2^chronic kidney disease was defined as structural or functional abnormalities of the kidneys for ≥3 months; ^3^breathing support included a simple face mask, nasal cannula, non-rebreathing mask, venturi mask, CPAP, BiPAP, or intubation.

## Data Availability

All data analyzed during this study are included in this published article and the accompanying supplementary material.

## Supplementary Materials

Supplementary Materials The supplementary tables and figures can be found in the supplementary material in the following order. Supplementary Table 1: Sequential Organ Failure Assessment (SOFA) score. Supplementary Table 2: Quick Sequential Organ Failure Assessment (qSOFA) score. Supplementary Table 3: Systemic Inflammatory Response Syndrome (SIRS). Supplementary Table 4: baseline characteristics of the cohort. Supplementary Figure 1: correlation between SOFA scores and SIRS score at admission. Supplementary Table 5: univariate logistic regression of demographic, clinical, and laboratory variables. Supplementary Table 6: AUROC analysis for different scores at admission. Supplementary Table 7: assessment of statistically significant differences in the predictive ability of mSOFA, SOFA, qSOFA, and SIRS. Supplementary Figure 2: receiver operating characteristic curves for different SOFA score derivatives. Supplementary Table 8: comparison between scores in predicting mortality in ICU at admission in different groups of patients.
