# Supplementary material for: Investigating the Utility of the SOFA Score and Creating a Modified SOFA Score for Predicting Mortality in the Intensive Care Units in a Tertiary Hospital in Jordan
Source: Crit Care Res Pract. 2023 Aug 7;2023:3775670. doi: 10.1155/2023/3775670 (PMC10425253; doi:10.1155/2023/3775670)
Supplement: Supplementary Materials — The supplementary tables and figures can be found in the supplementary material in the following order. Supplementary Table 1: Sequential Organ Failure Assessment (SOFA) score. Supplementary Table 2: Quick Sequential Organ Failure Assessment (qSOFA) score. Supplementary Table 3: Systemic Inflammatory Response Syndrome (SIRS). Supplementary Table 4: baseline characteristics of the cohort. Supplementary Figure 1: correlation between SOFA scores and SIRS score at admission. Supplementary Table 5: univariate logistic regression of demographic, clinical, and laboratory variables. Supplementary Table 6: AUROC analysis for different scores at admission. Supplementary Table 7: assessment of statistically significant differences in the predictive ability of mSOFA, SOFA, qSOFA, and SIRS. Supplementary Figure 2: receiver operating characteristic curves for different SOFA score derivatives. Supplementary Table 8: comparison between scores in predicting mortality in ICU at admission in different groups of patients. [file 3775670.f1.docx]

# Investigating the utility of the SOFA score and creating a modified SOFA score for predicting mortality in the intensive care units in a tertiary hospital in Jordan

Anas H. A. Abu-Humaidan^1ϯ*^, Fatima M. Ahmad ^ϯ 1,2^, Laith S Theeb^1^, Abdelrahman J. Sulieman^1^, Abdelkader Battah^1^, Amjad Bani Hani^3^, Mahmoud Abu Abeeleh^3^

1. Department of Pathology, Microbiology and Forensic Medicine, School of Medicine, The University of Jordan, Amman, Jordan.
2. Department of Clinical Sciences, School of Science, The University of Jordan, Amman, Jordan.
3. Department of General Surgery, Section of Cardiovascular Surgery, Jordan University Hospital, Amman, Jordan.

**^ϯ^** Both contributed equally to this work.

*Address correspondence to:

Anas Abu-Humaidan M.D. Ph.D.

E-mail: A.abuhumaidan@ju.edu.jo

Tel. number: +962779227922

### Supplementary Table 1. Sequential Organ Failure Assessment (SOFA) Score.

| **Score** | **0** | **1** | **2** | **3** | **4** |
| --- | --- | --- | --- | --- | --- |
| **System** | | | | | |
| **Respiratory** |  |  |  |  |  |
| PaO2 / FiO2, mmHg (kPa) | ≥400 (53.3) | <400 (53.3) | <300 (40) | <200 (26.7) with respiratory support | <100 (13.3) with respiratory support |
| **Coagulation** | | | | | |
| Platelet count × 103 / μL | ≥ 150 | < 150 | < 100 | < 50 | < 20 |
| **Liver** | | | | | |
| Bilirubin, mg / dL | <1.2 | 1.2–1.9 | 2.0–5.9 | 6.0–11.9 | >12.0 |
| **Cardiovascular** |  |  |  |  |  |
|  | MAP * ≥70 mm Hg | MAP <70 mm Hg | Dopamine <5 or dobutamine (all doses) | Dopamine 5.1–15 or epinephrine ≤0.1 or norepinephrine ≤0.1 | Dopamine>15 or epinephrine >0.1 or norepinephrine >0.1 |
| **Central Nervous System** | | | | | |
| Glasgow Coma Scale | 15 | 13–14 | 10–12 | 6–9 | < 6 |
| **Renal** | | | | | |
| Creatine, mg/dL | <1.2 | 1.2–1.9 | 2.0–3.4 | 3.5–4.9 | >5.0 |
| Urine Output |  |  |  | < 500 | < 200 |

### Supplementary Table 2. Quick Sequential Organ Failure Assessment (qSOFA) score.

| **qSOFA (Quick SOFA) Criteria** | **Points** |
| --- | --- |
| Respiratory rate ≥22/min | 1 |
| Change in mental status | 1 |
| Systolic blood pressure ≤100 mmHg | 1 |

### Supplementary Table 3. Systemic Inflammatory Response Syndrome (SIRS).

|  | |
| --- | --- |
| **SIRS is defined by the satisfaction of any two of the criteria below:** |  |
| 1. Body temperature over 38 or under 36 degrees Celsius. |  |
| 2. Heart rate greater than 90 beats/minute |  |
| 3. Respiratory rate greater than 20 breaths/minute or partial pressure of CO2 less than 32 mmHg |  |
| 4. Leukocyte count greater than 12,000 or less than 4,000 /microliters or over 10% immature forms or bands. |  |

### Supplementary Table 4. Baseline characteristics of the cohort.

|  | **Cohort (n= 194)** | | **Survivors (n= 159)** | | **Non-survivors (n= 35)** | |  |  |  |
| --- | --- | --- | --- | --- | --- | --- | --- | --- | --- |
| **Characteristics** | Mean ± SD (median) or N (%) | | | Mean ± SD (median) or N (%) | Mean ± SD (median) or N (%) | | p-value^1^ | |  |
| **Age** | 59.92 ± 16.7 (62.0) | | | 58.76 ± 16.6 (61.0) | 65.20 ± 13.6 (67.0) | | **<0.001** | |  |
|  |  | | |  |  | |  | |  |
| **Vitals** |  | | |  |  | |  | |  |
| Heart rate (beat/minute) | 88.2 ± 18.1 (88) | | | 87.3 ± 17.3 (88) | 92.4 ± 21.2 (85) | | 0.226 | |  |
| Temperature (℃) | 36.7 ± 0.7 (36.7) | | | 36.7 ± 0.7 (36.6) | 37.0 ± 0.6 (37.0) | | **<0.001** | |  |
| Respiratory rate (breaths/minute) | 20.1 ± 4.8 (20) | | | 20.3 ± 4.7 (20) | 19.2 ± 5.5 (20) | | 0.210 | |  |
| Systolic BP (mmHg) | 127.6 ± 21.8(125) | | | 129.4 ± 20.2(130) | 119.5 ± 26.9 (115) | | **0.014** | |  |
| Diastolic BP (mmHg) | 73.9 ± 15.6 (75) | | | 74.9 ± 15.2 (75) | 68.9 ± 16.8 (65) | | **0.036** | |  |
| MAP (mmHg) | 91.6 ± 16.5 (92) | | | 92.7 ± 15.7 (93) | 86.3 ± 19.3 (82) | | **0.037** | |  |
|  |  | | |  |  | |  | |  |
| **Lab Tests** |  | | |  |  | |  | |  |
| Haemoglobin, g/dL | 11.6 ± 2.3 (11.7) | | | 11.7 ± 2.4 (12) | 11.3 ± 2.1 (11) | | 0.308 | |  |
| PCV, % | 35.6 ± 6.6 (35.9) | | | 35.6 ± 6.7 (36.2) | 35.3 ± 6.3 (34) | | 0.791 | |  |
| WBC count 10^9^/L | 20.9 ± 110.4 (12.1) | | | 22.4 ± 121.9 (11.8) | 14.1 ± 7.8 (13) | | 0.482 | |  |
| Neutrophils (N) 10^9^/L | 80.4 ± 12.1 (83.2) | | | 79.5 ± 12.8 (82.7) | 84.3 ± 6.7 (84.3) | | 0.070 | |  |
| Lymphocytes (L) 10^9^/L | 13.1 ± 9.65 (10) | | | 13.8 ± 10.22 (10.2) | 10 ± 5.56 (9.7) | | 0.109 | |  |
| N/L ratio | 10.9 ± 9.96 (8.3) | | | 10.7 ± 10.39 (7.6) | 11.8 ± 7.8 (8.7) | | 0.094 | |  |
| Platelets 10^9^/L | 278.5 ± 133.92 (259.5) | | | 283.4 ± 127.33 (270) | 255.8 ± 160.7 (232) | | 0.065 | |  |
| Bilirubin | 1 ± 1.46 (0.6) | | | 0.9 ± 1.25 (0.6) | 1.4 ± 2.12 (0.7) | | 0.052 | |  |
| Total protein (g/dl) | 6 ± 0.97 (6) | | | 6.1 ± 0.99 (6) | 5.7 ± 0.83 (5.7) | | 0.052 | |  |
| Creatinine, mg/dl | 1.2 ± 1.43 (0.8) | | | 1 ± 1.06 (0.7) | 2.3 ± 2.25 (1.9) | | **<0.001** | |  |
| Sodium, mmol/L | 137.4 ± 4.75 (138) | | | 137.2 ± 4.61 (138) | 138.4 ± 5.29 (139) | | 0.169 | |  |
| Potassium, mmol/L | 4.3 ± 0.71 (4.3) | | | 4.3 ± 0.58 (4.3) | 4.6 ± 1.11 (4.4) | | 0.345 | |  |
| Chloride, mmol/L | 101.9 ± 9.44 (103) | | | 102.5 ± 5.94 (103) | 99.3 ± 18.26 (102) | | 0.645 | |  |
| CRP0 | 109.2 ± 117.27 (61.9) | | | 102.5 ± 111.81 (56.1) | 134.7 ± 134.9 (84.6) | | 0.241 | |  |
| RBS, mg/dl | 167.4 ± 79.46 (149) | | | 162.4 ± 72.83 (145) | 190.5 ± 102.59 (154) | | 0.096 | |  |
| PaO2 | 104.4 ± 49.62 (90.4) | | | 102.3 ± 46.65 (89.1) | 113.8 ± 60.98 (95) | | 0.315 | |  |
| FiO2 | 0.6 ± 2.77 (0.3) | | | 0.7 ± 3.06 (0.3) | 0.5 ± 0.29 (0.4) | | **0.009** | |  |
| Pa/FiO2 Ratio | 320.5 ± 163.16 (310.5) | | | 328.5 ± 165.76 (318.1) | 283.9 ± 147.48 (286.5) | | 0.078 | |  |
| **Comorbidities** |  | | |  |  | |  | |  |
| Cancer | 43 (22.2%) | | | 33 (20.8%) | 10 (28.6%) | | 0.313 | |  |
| Kidney injury | 40 (20.6%) | | | 23 (14.5%) | 17 (48.6%) | | **<0.001** | |  |
| - *CKD* | 18 (9.3%) | | | 8 (5%) | 10 (28.6%) | | **<0.001** | |  |
| - *AKI* | 30 (15.5%) | | | 18 (11.3%) | 12 (34.3%) | | **0.001** | |  |
| - *ESRD* | 6 (3.1%) | | | 2 (1.3%) | 4 (11.4%) | | **0.013** | |  |
| - *Dialysis* | 9 (4.6%) | | | 3 (1.9%) | 6 (17.1%) | | **0.014** | |  |
| Heart disease | 70 (36.1%) | | | 52 (32.7%) | 18 (51.4%) | | **0.037** | |  |
| - *IHD* | 37 (19.1%) | | | 28 (17.6%) | 9 (25.7%) | | 0.269 | |  |
| - *Atrial fibrillation* | 23 (11.9%) | | | 15 (9.4%) | 8 (22.9%) | | **0.026** | |  |
| - *CAD* | 2 (1.0%) | | | 0 (0%) | 2 (5.7%) | | **0.032** | |  |
| - *HF* | 32 (16.5%) | | | 26 (16.4%) | 6 (17.1%) | | 0.909 | |  |
| - *CVD* | 1 (0.5%) | | | 1 (0.6%) | 0 (0%) | | 1.000 | |  |
| Diabetes | 93 (47.9%) | | | 71 (44.7%) | 25 (71.4%) | | 0.051 | |  |
| HTN | 111 (57.2%) | | | 86 (54.1%) | 25 (71.4%) | | 0.060 | |  |
|  |  | | |  |  | |  | |  |
| **Ventilation** | 150 (77.3%) | | | 119 (74.8%) | 31 (88.6%) | | 0.117 | |  |
|  |  |  |  | |  |  | |  | |

**^1^** p-values ≤ 0.05 are in bold**.**


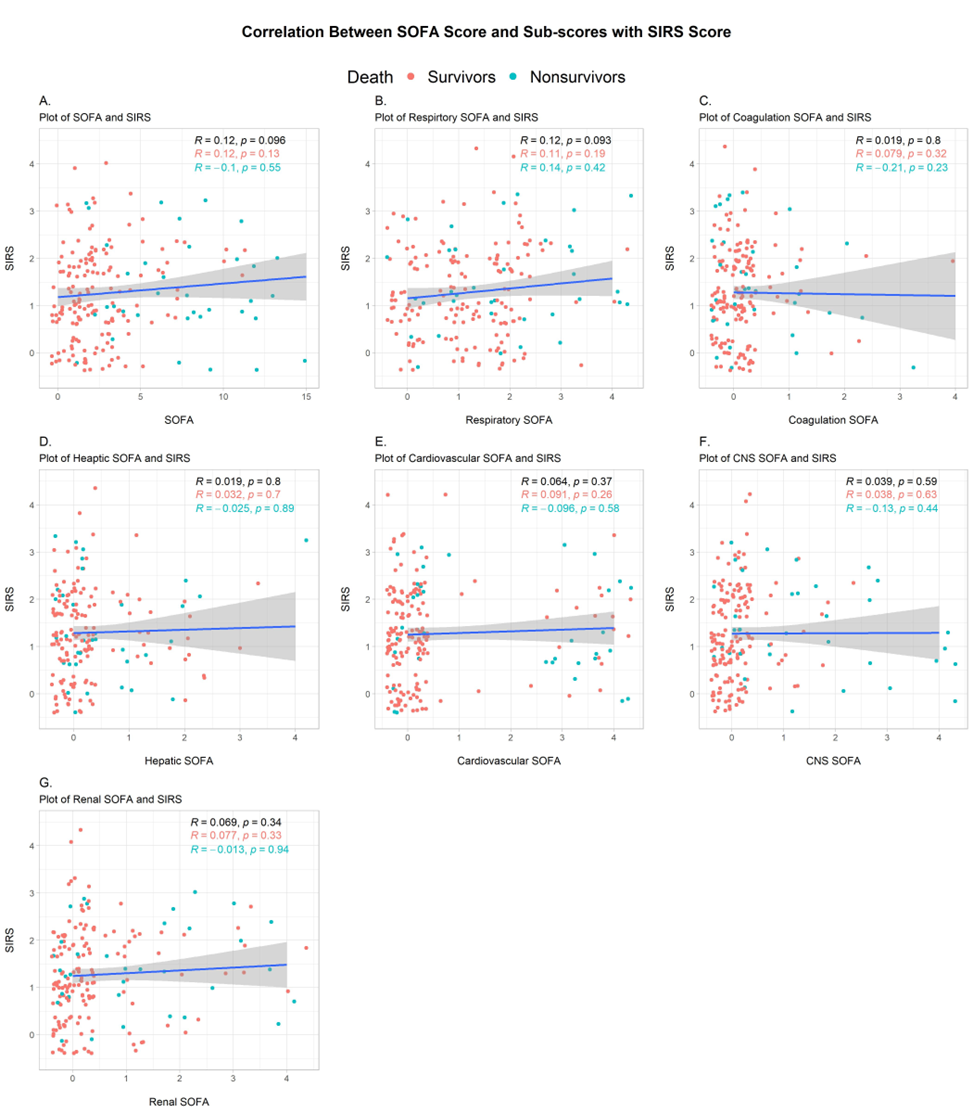


Supplementary Figure 1. Correlation between SOFA scores and SIRS score at admission. Spearman’s correlation coefficient (R) was used to investigate the correlation between SIRS criteria (scored 0-4) and (A) total SOFA score, (B) respiratory SOFA, (C) coagulation SOFA, (D) hepatic SOFA, (E) cardiovascular SOFA, (F) central nervous system SOFA, and (G) renal SOFA. Each dot represents the scores of one patient at admission. Red dots represent non-survivors while green dots represent survivors.

### Supplementary Table 5. Univariate logistic regression of demographic, clinical, and laboratory variables.

|  | **Univariate** | | |
| --- | --- | --- | --- |
| **Characteristics** | **OR** | **CI (95%)** | **p-value^1^** |
| **Age** |  |  |  |
| <60 | - | - | - |
| >=60 | 1.62 | 0.75-3.71 | 0.232 |
|  |  |  |  |
| **Vitals** |  |  |  |
| Heart rate (beat/minute) | 1.02 | 1.00-1.04 | 0.056 |
| Temperature (℃) |  |  |  |
| 37-38.3 | - | - | - |
| < 37 | 0.24 | 0.10-0.54 | **0.001** |
| >= 38.3 | 0.51 | 0.03-3.70 | 0.558 |
| Respiratory rate (breaths/minute) | 0.98 | 0.91-1.06 | 0.631 |
| Systolic BP (mmHg) | 0.98 | 0.96-1.00 | 0.079 |
| Diastolic BP (mmHg) | 0.98 | 0.95-1.00 | 0.096 |
| MAP (mmHg) | 0.98 | 0.95-1.00 | 0.104 |
|  |  |  |  |
| **Lab Tests** |  |  |  |
| Haemoglobin, g/dL | 0.94 | 0.79-1.10 | 0.432 |
| PCV, % | 1.00 | 0.94-1.05 | 0.881 |
| WBC count 10^9^/L | 1.00 | NA-1.00 | 0.720 |
| Neutrophils (N) 10^9^/L | 1.04 | 1.00-1.09 | 0.062 |
| Lymphocytes (L) 10^9^/L | 0.96 | 0.90-1.00 | 0.080 |
| N/L ratio | 1.00 | 0.96-1.04 | 0.876 |
| Platelets 10^9^/L | 1.00 | 1.00-1.00 | 0.281 |
| Bilirubin | 1.18 | 0.94-1.48 | 0.136 |
| Total protein (g/dl) | 0.69 | 0.45-1.02 | 0.067 |
| Creatinine, mg/dl | 1.56 | 1.23-2.09 | **0.001** |
| Sodium, mmol/L | 1.07 | 0.99-1.16 | 0.114 |
| Potassium, mmol/L | 1.70 | 1.04-2.81 | **0.034** |
| Chloride, mmol/L | 0.99 | 0.94-1.05 | 0.848 |
| RBS, mg/dl | 1.00 | 1.00-1.01 | 0.126 |
| PaO2 | 1.01 | 1.00-1.01 | 0.131 |
| FiO2 | 0.96 | NA-1.09 | 0.683 |
| Pa/FiO2 Ratio | 1.00 | 0.99-1.00 | 0.138 |
|  |  |  |  |
| **SOFA Organ-specific sub-components** |  |  |  |
| Respiratory | 1.84 | 1.27-2.74 | **0.002** |
| Coagulation | 1.93 | 1.11-3.48 | **0.022** |
| Hepatic | 1.66 | 1.05-2.62 | **0.028** |
| Cardiovascular | 1.64 | 1.30-2.08 | **0.001** |
| CNS | 5.20 | 2.92-10.61 | **0.001** |
| Renal | 1.96 | 1.43-2.76 | **0.001** |
|  |  |  |  |
| **Comorbidities** |  |  |  |
| Cancer | 1.52 | 0.63-3.48 | 0.329 |
| Kidney injury | 5.14 | 2.29-11.71 | **0.001** |
| *CKD* | 6.61 | 2.38-19.0 | **0.001** |
| *AKI* | 3.79 | 1.57-9.03 | **0.003** |
| *ESRD* | 8.87 | 1.65-66.12 | **0.014** |
| *Dialysis* | 9.43 | 2.34-46.83 | **0.002** |
| Heart disease | 1.81 | 0.85-3.89 | 0.125 |
| *IHD* | 1.58 | 0.63-3.73 | 0.304 |
| *Atrial fibrillation* | 2.66 | 0.98-6.90 | **0.047** |
| *CAD* | N/A | | |
| *HF* | 0.80 | 0.25-2.13 | 0.672 |
| *CVD* | N/A | | |
| Diabetes | 2.03 | 0.95-4.56 | 0.075 |
| HTN | 1.92 | 0.87-4.49 | 0.115 |
| Ventilation | 2.62 | 0.95-9.30 | 0.089 |

**^1^** p-values ≤ 0.05 are in bold**.**

### Supplementary Table 6. AUROC analysis for different scores at admission.

| **Score** | **AUROC^1^** | **95% CI** | **p-value^2^** | **Sensitivity (cut-off)** | **specificity** |
| --- | --- | --- | --- | --- | --- |
| mSOFA**^3^** | 0.894 | 0.835-0.952 | **<0.001** | 0.914 (2) | 0.766 |
| SOFA | 0.868 | 0.801-0.934 | **<0.001** | 0.886 (3) | 0.628 |
| qSOFA | 0.746 | 0.655-0.836 | **<0.001** | 0.829 (2) | 0.607 |
| SIRS**^4^** | 0.533 | 0.425-0.641 | 0.551 | 0.400 (2) | 0.593 |

**^1^** p-values ≤ 0.05 are in bold.

### Supplementary Table 7. Assessment of statistically significant differences in the predictive ability of mSOFA. SOFA, qSOFA, and SIRS

| **Score** | **vs. mSOFA** | **vs. SOFA** | **vs. qSOFA** | **vs. SIRS** |
| --- | --- | --- | --- | --- |
| mSOFA | ref | 0.4032 | **<0.001** | **< 0.001** |
| SOFA | 0.4032 | ref | **0.011** | **< 0.001** |
| qSOFA | **<0.001** | **0.011** | ref | **< 0.001** |
| SIRS | **< 0.001** | **< 0.001** | **< 0.001** | ref |

**^1^** The predictive ability of each score p-values ≤ 0.05 are in bold.


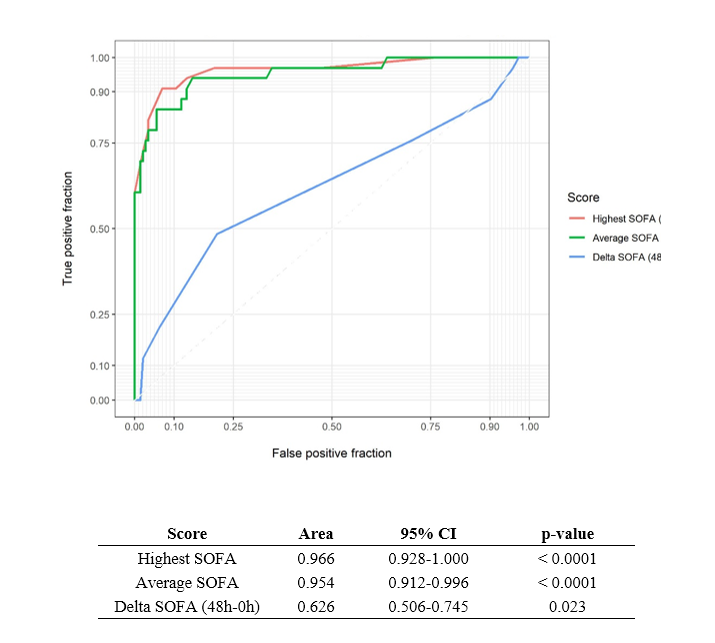
Supplementary Figure 2. Receiver operator characteristic curves for different SOFA score derivatives. Area under the receiver operator characteristic curve (AUROC) values, the 95% confidence intervals, and p-values for each score are displayed below the graph.

### Supplementary Table 8. Comparison between scores in predicting mortality in ICU at admission in different groups of patients.

| Heart disease | **vs. mSOFA** | **vs. SOFA** | **vs. qSOFA** | **vs. SIRS** |
| --- | --- | --- | --- | --- |
| mSOFA | ref | **0.033** | <0.001 | **<0.001** |
| SOFA | **0.033** | ref | 0.390 | **0.003** |
| qSOFA | **<0.001** | 0.390 | ref | **0.001** |
| SIRS | **<0.001** | **0.003** | **0.001** | ref |
|  |  |  |  |  |
| Kidney disease | **vs. mSOFA** | **vs. SOFA** | **vs. qSOFA** | **vs. SIRS** |
| mSOFA | ref | 0.820 | **0.025** | **0.001** |
| SOFA | 0.820 | ref | 0.092 | **0.003** |
| qSOFA | **0.025** | 0.092 | ref | 0.145 |
| SIRS | **0.001** | **0.003** | 0.145 | ref |
|  |  |  |  |  |
| Hypertension | **vs. mSOFA** | **vs. SOFA** | **vs. qSOFA** | **vs. SIRS** |
| mSOFA | ref | 0.558 | **0.001** | **<0.001** |
| SOFA | 0.558 | ref | **0.006** | **<0.001** |
| qSOFA | **0.001** | **0.006** | ref | **<0.001** |
| SIRS | **<0.001** | **<0.001** | **<0.001** | Ref |
|  |  |  |  |  |
| Diabetes | **vs. mSOFA** | **vs. SOFA** | **vs. qSOFA** | **vs. SIRS** |
| mSOFA | ref | 0.219 | **<0.001** | **<0.001** |
| SOFA | 0.219 | ref | 0.009 | **<0.001** |
| qSOFA | **<0.001** | **0.009** | ref | **0.002** |
| SIRS | **<0.001** | **<0.001** | **0.002** | ref |

**^1^** p-values ≤ 0.05 are in bold**.**
